# Significant reductions in tertiary hospital encounters and less travel for families after implementation of Paediatric Care Coordination in Australia

**DOI:** 10.1186/s12913-018-3553-4

**Published:** 2018-10-03

**Authors:** Christie Breen, Lisa Altman, Joanne Ging, Marie Deverell, Susan Woolfenden, Yvonne Zurynski

**Affiliations:** 1 Integrated Care Program, Sydney Children’s Hospitals Network, Sydney, Australia; 2 Discipline of Child and Adolescent Health, Sydney Medical School, The University of Sydney, Sydney, Australia; 3 Australian Paediatric Surveillance Unit, Kids Research Institute, the Children’s Hospital at Westmead, Sydney, Australia; 4 Integrated Care Program and Department of Community Child Health, Sydney Children’s Hospitals Network, Sydney, Australia; 5 School of Women’s and Children’s Health, Faculty of Medicine, University of New South Wales, Sydney, Australia; 6 Australian Institute of Health Innovation, Faculty of Medicine and Health Sciences, Macquarie University, Sydney, Australia

**Keywords:** Children with medical complexity, Care coordination, Integrated care, Models of care, Chronic disease

## Abstract

**Background:**

Over a third of Australian children have long-term health conditions, often involving multiple organ systems and resulting in complex health care needs. Our healthcare system struggles to meet their needs because of sectoral fragmentation and episodic models of care. Children with medical complexity (CMC) currently rely on tertiary paediatric hospitals for most of their healthcare, but this is not sustainable. We evaluated the impacts of Care Coordination on tertiary hospital service use and family outcomes.

**Methods:**

A pre- and post-implementation cohort evaluation of the Care Coordination service at a tertiary paediatric hospital network, was undertaken. From July 2015 CMC enrolled in the service had access to a Care Coordinator, shared-care plans, linkage with local general practitioners (GPs), and access to a 24-h Hotline from August 2016. CMC were those with ≥4 emergency department (ED) presentations, hospital stays of ≥14 days, or ≥ 10 outpatient appointments in 12 months. Medically fragile infants at risk of frequent future hospital utilisation, and children with medical problems complicated by difficult family psychosocial circumstances were also included. Care Coordinators collected outcomes for each enrolled child. Administrative data on hospital encounters 6 months pre- and post-enrolment were analysed for children aged > 6 months.

**Results:**

An estimated 557 hospital encounters, were prevented in the 6 months after enrolment, for 534 children aged > 6 months. ED presentations decreased by 40% (Chi^2^ = 37.95; *P* < 0.0001) and day-only admissions by 42% (Chi^2^ = 7.54; *P* < 0.01). Overnight admissions decreased by 9% but this was not significant. An estimated Au$4.9 million was saved over 2 years due to prevented hospital encounters. Shared-care plans were developed for 83.5%. Of 84 children who had no regular GP, 58 (69%) were linked with one. Fifty-five (10%) of families were linked to the 24-h Hotline to enable remote access to support and advice. Over 50,000 km of family travel and 370 school absences was prevented.

**Conclusions:**

The Care Coordination service has clear benefits for the tertiary paediatric hospital network and for families. Ongoing evaluation is essential for continuous improvement and to support adjustments to the model according to the local context.

## Background

The Australian Institute of Health and Welfare (AIHW), has estimated that approximately 37% of Australian children have at least one long-term health condition, and the number of children with medical complexity (CMC) is increasing [1]. The long-term conditions refer to conditions that last 6 months or more, or are expected to last 6 months or more [1]. Most of these children have asthma, allergy or diabetes, however, children with a wide variety of diagnoses, which often involve multiple organ systems, and ongoing and complex health care needs, are known to access hospital, specialist and primary care services frequently [2]. Although there is no universal consensus as to what constitutes complex health care needs, CMC may have developmental and behavioural problems, intellectual and physical disabilities, and many are reliant on specialised medical equipment and appliances, and require frequent specialised care from multidisciplinary teams [3, 4]. Care provided at tertiary paediatric hospitals ensures access to multiple specialists, however, CMC also access care in the community from many different health care providers and welfare services [4, 5].

The Australian health system is complex [6, 7]. Australia has universal health care coverage through the Medical Benefits Scheme or Medicare and the Pharmaceutical Benefits Scheme [6]. There is a general lack of integration between the primary health care sector and the hospital sector, and this is further complicated by a split in funding and responsibilities between the federal and state and territory governments, and a complex system of re-imbursements and subsidies [6]. Services may be provided by private providers or private hospitals as well as publicly funded hospitals and community health services. Approximately 50% of Australians choose to participate in private health insurance schemes which are subsidised by the Australian Government through Medicare [6]. The OECD concluded that: “…the Australian healthcare system is too complex for patients” especially for patients with chronic conditions [7].

Our health system struggles to meet the complex needs of CMC because it is designed for episodic care largely provided by health care professionals who are highly specialised to single organ systems, and are accustomed to working in “silos” defined by their medical specialties, institutions, geographical areas and informal professional networks [8, 9]. Parents and caregivers of CMC find care navigation difficult, because of the disconnectedness and complexity of our healthcare system [10]. This, on top of providing care to their sick child, often results in emotional and financial strain [4]. Families often experience the burden of repeated travel, family disruption and out-of-pocket expenses when accessing health care though a major paediatric hospital and these burdens are further amplified for families who live in regional or remote areas or those who live with psychosocial complexity and isolation [11]. A survey of parents/carers attending a tertiary children’s outpatient department in Sydney indicated that 44% travelled three to nine hours to reach the hospital and this resulted in financial burden, time away from paid employment and school absences [11, 12]. This limits opportunities to participate in school and social activities and also has an impact on the family, as parents or caregivers reduce hours of paid employment to ensure that their child accesses essential health care [13]. Recently, the productivity cost for families who have a child admitted to hospital, was estimated at Au$589 per patient day [11].

Linking tertiary hospital healthcare with community-based healthcare may be cost effective while meeting healthcare needs for CMC, however if this is not facilitated and coordinated the burden of navigating care becomes the responsibility of the family [14]. In the USA, the cost of uncoordinated care was estimated to be up to 35% higher than cost of coordinated care [14]. The World Health Organisation (WHO), the Organisation for Economic Cooperation and Development (OECD) and the Australian Productivity Commission have all advocated for integrated care services that are joined-up, easy to navigate and minimise the number of separate visits required to get the needed care [7, 15, 16]. The WHO advocates for integrated people-centred health service models and systems that support health sector and inter-sectoral integration including strong integration of primary care, care coordination, responsiveness to needs of individuals and support for self-care, quality and safety and equity within a holistic framework [17]. A rapid review of the literature showed an increasing evidence base to support the implementation of integrated care models and care coordination for CMC [18]. Team based, multi-agency support is advocated as best practice for CMC, and this approach is favoured by families [19]. Health care coordination has been shown to support families when navigating complex health systems while avoiding service duplication, supporting best practice, and improving health outcomes [20, 21].

Published evidence of the effectiveness and utility of integrated care initiatives in the Australian context is limited. One paper describing the Ambulatory Care Coordination program in Western Australia reported reductions in the number of hospital admissions, length of stay and emergency presentations for children with complex health needs and resulted in an estimated Au$1.9million in cost savings [22]. We found no other published papers describing the outcomes and impacts of paediatric integrated care.

In this paper we report implementation and evaluation outcomes of the Care Coordination service implemented in July 2015 at a large tertiary paediatric hospital network in Sydney, Australia.

## Methods

We studied changes to hospital utilisation and associated health care costs 2 years after implementation of the program. We also aimed to estimate benefits for the family in terms of saved travel time and school absences. The study design was a longitudinal pre- and post-implementation cohort evaluation of the Care Coordination service.

### Setting

The Sydney Children’s Hospitals Network (SCHN) incorporates two large tertiary paediatric hospitals in New South Wales (NSW), Australia - The Children’s Hospital at Westmead (CHW) and the Sydney Children’s Hospital (SCH). SCHN is the largest paediatric health service provider in Australia and provides approximately 90% of all tertiary paediatric care in NSW. In the financial year June 2015 to July 2016, there were 50,474 admissions, 23,467 day-only admissions, 1,124,158 outpatient encounters and 96,288 ED presentations at the SCHN [23].

### Intervention: The Kids Guided Personalised Service (Kids GPS) care coordination program

**Fig. 1:**
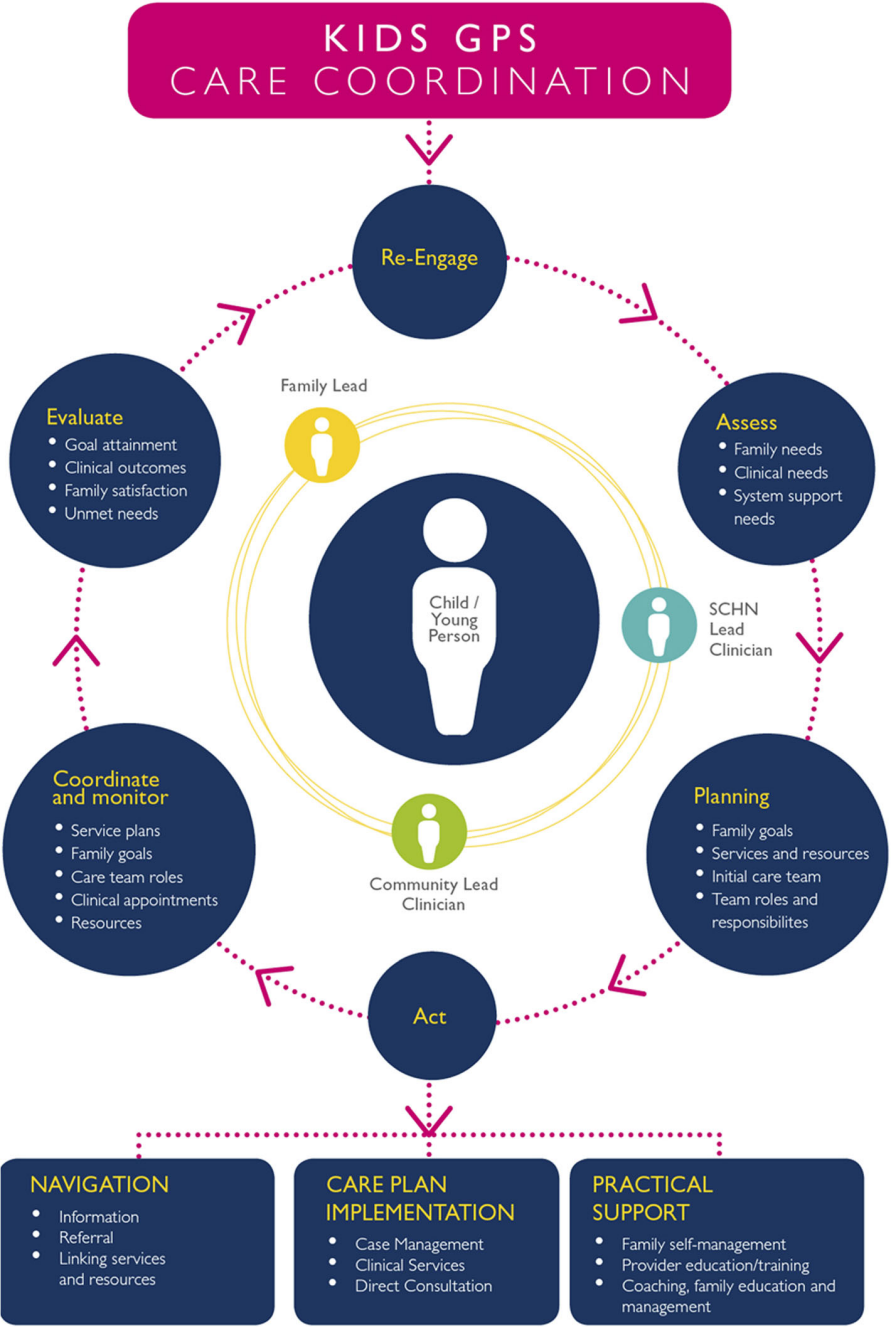
Circle of Coordination framework that underpins the Kids GPS Care Coordination service *(*Adapted from Cohen et al. [4])

Care Coordinators are essential to the Kids GPS Care Coordination Program, and they help to build a ‘Circle of Coordination’ that places the child at the centre, (Fig. 1) [5]. The circle is formed by involving ‘lead’ persons within SCHN, the local community health services and the family, all of whom share the responsibility to ensure active communication among all involved in the health care needs of the child. The Kids GPS Care Coordination service was fully implemented at SCHN in July 2015.

The four Care Coordinators at SCHN work with individual families to understand their goals and inform the development of the most appropriate shared-care plan. They also work closely with treating teams no matter where they are located, including in the community or in local hospitals, to identify opportunities for shared care and to move care closer to the families’ home. Support through education, networking and partnerships empowers families and local health care providers to manage the child in the community to reduce ED presentations and hospitalisations.

The Kids GPS service targeted children with complex health or psychosocial needs according to the criteria in Table 1. Children already receiving care coordination from their medical team were excluded to avoid duplication of service, however, the care coordinators assisted these teams when needed.

**Table 1:** Eligibility criteria for Care Coordination enrollment and three-tiered classification of complexity of coordination

Eligibility:	
Children aged 0–19 years who were patients of the SCHN and:	
1. Had complex needs, involving multiple health care providers and health services	
2. There was no designated key person already coordinating care within a multidisciplinary team	
3. There was potential to facilitate a more coordinated approach to the patient’s healthcare needs, in particular for patients who frequently used hospitals services including:	
a) More than 4 ED presentations within 12 months;	
b) More than 14 days length of stay for any hospitals admission;	
c) More than 10 outpatient appointments within the last 12 months;	
d) Infants who were medically fragile and identified as being at risk of significant future hospital utilization.	
Care coordination complexity levels:	
• Tier-1: Integration with primary care only	
• Tier-2: Requires integration and shared care plan +/− primary care	
• Tier-3: Chronic/complex care coordination among tertiary, local health district and primary care	

At the beginning of service implementation, eligible children were retrospectively identified by analysing medical records according to eligibility criteria. In the second year a routine algorithm was set up to analyse electronic medical records and to prospectively identify children who met eligibility criteria each week. In addition, medical teams or individual health care providers referred patients to the Care Coordination service.

### Key performance indicators and measures

During the design phase, targets for the number of enrolments were set by benchmarking against other similar paediatric coordination services including the Ambulatory Care Service at Princess Margaret Hospital in Perth, Western Australia [22] and the Care Coordination Service at the Royal Children’s Hospital in Melbourne, Victoria. A caseload of approximately 50 patients per full time equivalent (FTE) care coordinator was thought to be manageable. Therefore, as care coordinator capacity increased so did the targets: 100 enrolled children at 2 FTE, 150 at 3 FTE and 200 enrolled at full capacity of 4 FTE. Targets were established as ‘set and test’ measures, allowing increases or decreases when a clearer understanding of service capabilities and the workload became apparent.

### Data collection

Demographic data including the child’s age, diagnosis and post code was collected by care coordinators. Reasons for enrolment in the service and complexity of coordination required according to the three-tiered classification were also collected. Care coordinators prospectively collected outcome measures from the beginning of implementation including:

- Number of children referred and accepted into the Care Coordination Service
- Number of care plans developed and shared across teams and with families
- Number of patients linked to a GP after enrolment in the service
- Estimated number of ED presentations, overnight admissions, day only admissions and outpatient appointments avoided
- Care being received closer to home and savings measured in terms of occasions of travel and distance of travel, and school absences
- Number of families linked with the 24-h Hotline.

The Care Coordinators deemed an encounter as avoided in the following circumstances:

- if regular appointments at SCHN were no longer needed because they could be delivered closer to home;
- multiple appointments were streamlined and coordinated for a single day to avoid travel and school and work absences;
- ED presentations were avoided because of a care plan that empowered the family and local providers;
- ED presentations were avoided because direct and timely access to an Acute Review Clinic was established for the patient
- Admissions were prevented because of shifting of routine care to the hospitals in the local health district (LHD) or to primary care, ensuring that the patient received optimum care to avoid crisis.

In addition, data on the number of admissions and ED presentations 6 months before enrolment and 6 months after enrolment in the care coordination service was extracted from routine administrative data collections of the SCHN Management Analysis and Support Unit (MSAU). Babies aged < 6 months of age at the time of enrolment were excluded from calculations because the shorter pre-enrolment period would have biased the results. Any child that died during the post-enrolment period was also excluded from analysis.

### Analysis

Descriptive statistics (frequencies, mean, median standard deviation and range) were used to describe the enrolled cohort of patients. Comparisons of proportions across groups were analysed using Chi-square tests. Generalised estimating equations were used to examine the association between numbers of admissions and ED presentations in the six-month periods before and after enrolment in the Care Coordination service. These models assumed a Poisson distribution and allowed for adjustment for correlation between the outcomes in the same patient.

The family’s postcode enabled classification according to the Australian Index of Relative Socio-Economic Advantage and Disadvantage (IRSAD) [24]. The IRSAD summarizes information about the economic and social conditions of people and households within a geographical area defined by postal codes, and reflects relative advantage and disadvantage, on a scale from 0 (most disadvantaged) to 10 (most advantaged) [24].

A detailed economic analysis was outside of the scope of this study, however, simple cost savings were estimated based on average costs per encounter as calculated by the MSAU at SCHN. Travel distances saved for families were estimated based on the distance (in kilometres) between their residential address and the children’s hospital (SCH or CHW) and the number of encounters prevented as recorded prospectively by care coordinators. The costs associated with travel were estimated based on the whole of vehicle life per kilometre costs for common vehicles (e.g. Ford Mondeo) published by the NRMA (National Roads and Motorists Association) and the average cost of petrol per kilometer.

### Ethical approval

Ethical approval was obtained through SCHN Human Research Ethics Committee (approval number: LNR/15/SCHN/299).

## Results

**Fig. 2:**
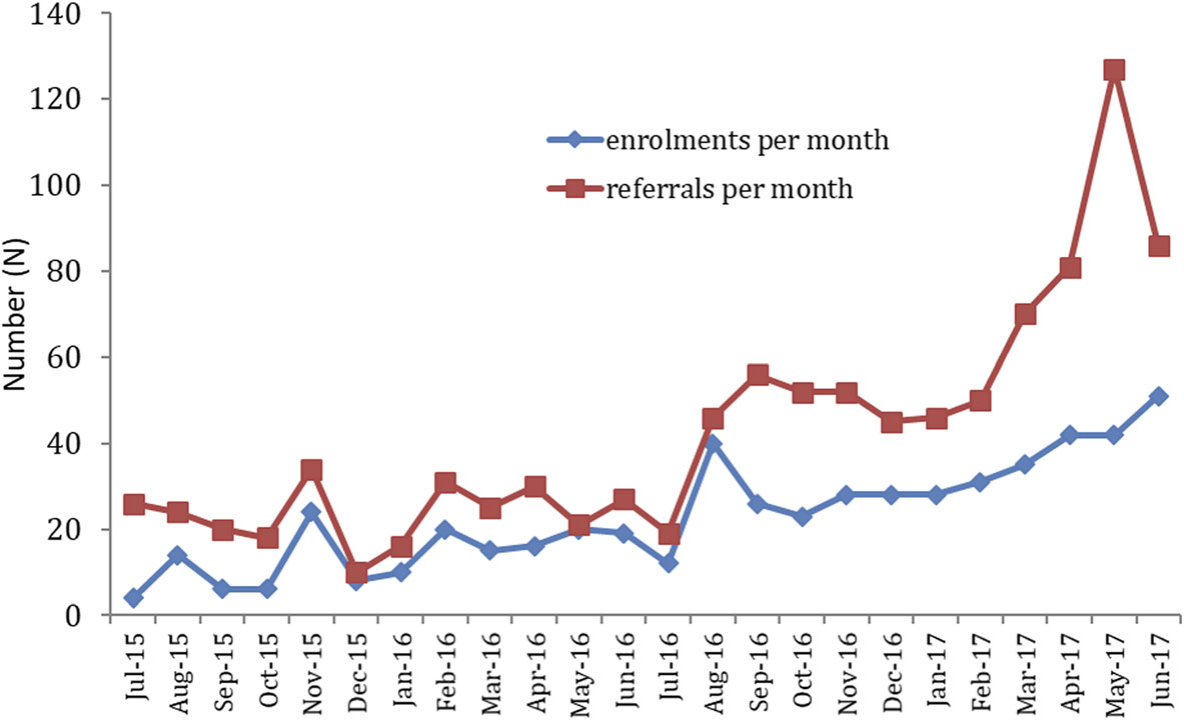
Number of new referrals and active enrolments being managed by the Coordination Service

There were 1004 referrals to the Kids GPS Care Coordination service at SCHN and 534 children were enrolled. The number of new referrals more than doubled during the second year: 281 referrals in year 1 and 723 referrals in year 2; suggesting increasing awareness and demand for the service. The number of active enrolments continued to increase, and the target of 50 enrolled children managed by each coordinator was exceeded in the second year. There was a wide gap between the number of referrals and enrolments, particularly during the second year (Fig. 2). The reasons for non-enrolment during year 2 included ineligible patients who did not meet inclusion criteria (*N* = 245), patients who were discharged or died (*N* = 155), and 99 patients were referred to another, more suitable service without enrolment in the coordination service.

Enrolled children had a median age of 5 years, (range: 7–19 years) and approximately 43% lived in socio-economically disadvantaged areas (IRSAD < 5). Of the 534 children enrolled, 446(83.5%) had a shared care plan implemented, while the other 88 children included those who already had a current plan managed by the child’s medical team but needed additional support, or they required appointment coordination only. Of the 84 families who did not have a regular GP before enrolment, 58(69%) were linked with their local GP after enrolment. The 24-h Hotline was introduced in late 2016 and 55(10.3%) patients were linked with the Hotline service by June 2017.

### Diagnoses and hospital teams involved

Children with genetic or chromosomal disorders were the most commonly referred for care coordination, followed by children with poorly controlled asthma, gastrointestinal disorders and congenital birth defects (Table 2). In addition to the main conditions listed in Table 2, enrolled children had many other conditions including immunological or rheumatological disorders, seizure disorders, and serious injuries. Children were mostly referred by general medicine teams, neurology, gastroenterology, rehabilitation, cardiology, immunology, orthopaedics and respiratory medicine, (Table 2), however, an even wider variety of teams and specialists were involved in the care of these children.

**Table 2:** Most common diagnostic groups and the principal medical teams involved in care of enrolled children

Diagnostic groups	N (%)
Genetic or Chromosomal disorder	101(18.9)
Asthma	86(16.1)
Gastro-intestinal Disorder	40(7.4)
Congenital defects (including congenital heart)	32(6.0)
Cerebral Palsy	18(3.4)
Developmental Delay	15(2.8)
Chronic Respiratory Disorder (not Asthma)	12(2.2)
Principal Teams Involved in Care	
General Medicine	126(23.6)
Gastroenterology	46(8.6)
Rehabilitation	24(4.5)
Neurology	35(6.6)
Cardiology	16(3.0)
Rheumatology	9(1.7)

### Level of coordination needed

Of all the children enrolled across SCHN, the majority (44.7%) needed coordination with primary care (Tier-1), while 26.8% required Tier-2 coordination with a shared care plan and integration with primary care, and 28.5% needed more complex multi-sector Tier-3 coordination.

### Impact on SCHN services

According to routine administrative data extracted by the MSAU at SCHN for enrolled children aged 6 months or older, day-only admissions decreased significantly (by 42%) in the 6-month period after enrolment when compared with the six-month period before enrolment, (Table 3). ED presentations also decreased significantly (by 40%), but the 9% decrease in overnight admissions was not statistically significant (Table 3). Based on these hospital administrative data, it is estimated that 557 encounters including 49 overnight admissions, 200 ED presentations, and 308 day-only admissions were saved at SCHN over the 6 months after enrolment among the 452 children aged 6 months or more. There were no outpatient department presentations recorded in the hospital administrative data before or after enrolment for these children.

**Table 3:** Comparison of hospital encounters 6 months before and after enrolment

	Pre-enrolment Encounters (N)	Post-enrolmentEncounters (N)	Rate Ratio	95% CI	Chi^2^	*P*-value
Admissions (over-night)	489	440	0.91	0.74–1.12	0.84	0.36
Admissions (day-only)	473	273	0.58	0.41–0.80	7.54	0.006
ED Presentations	777	469	0.61	0.52–0.70	37.95	< 0.0001

Based on average per encounter costs calculated by the MSAU for financial year 15/16 and financial year 16/17, the total cost savings for admissions and ED presentations for the 453 children aged more than 6 months, was $1,226,079 6 months post enrolment and an estimated $4,904,316 over 2 years. (Table 4). Data collected prospectively by care coordinators over 2 years after implementation of the Care Coordination service estimated that 876 encounters were prevented for the total of 543 children enrolled. The care coordinators estimated that 290 hospitalizations were prevented in addition to 204 day-only admissions and 312 ED presentations. Using the same per encounter average costs and the data collected by the care coordinators, the savings were estimated at Au$4,526,286 over 2 years, (Table 4).

**Table 4:** Estimated encounter and cost savings after enrolment in the service

Encounter	Average cost per encounter at SCHN	Based on administrative data from the MSAU^a^			Based on data collected by the Care Coordinators^b^	
		Actual Encounters saved 6 months after enrolment	Total amount saved 6 months after enrolment	Estimated amount saved over 2 years	Estimated encounters saved over 2 years	Estimated amount saved over 2 years
	(Au$)	(N)	(Au$)	(Au$)	(N)	(Au$)
Admissions (over-night)	12,927	49	633,423	2,533,692	290	3,748,830
Admissions (day-only)	2144	200	428,800	1,715,200	312	668, 928
ED Presentations	532	308	163,856	655,424	204	108,528
Total Estimated savings (2 yrs)	$4,904,316				$4,526,286	

^a^These cost estimates are for 453 children aged over 6 months, where the number of encounters 6 months before and after enrolment were compared to estimate the number of encounters saved

^b^These cost are estimated based on data collected prospectively by care coordinators for all children enrolled in the service over 2 years after implementation

### Benefits for families: travel saved and school absences avoided

**Fig. 3:**
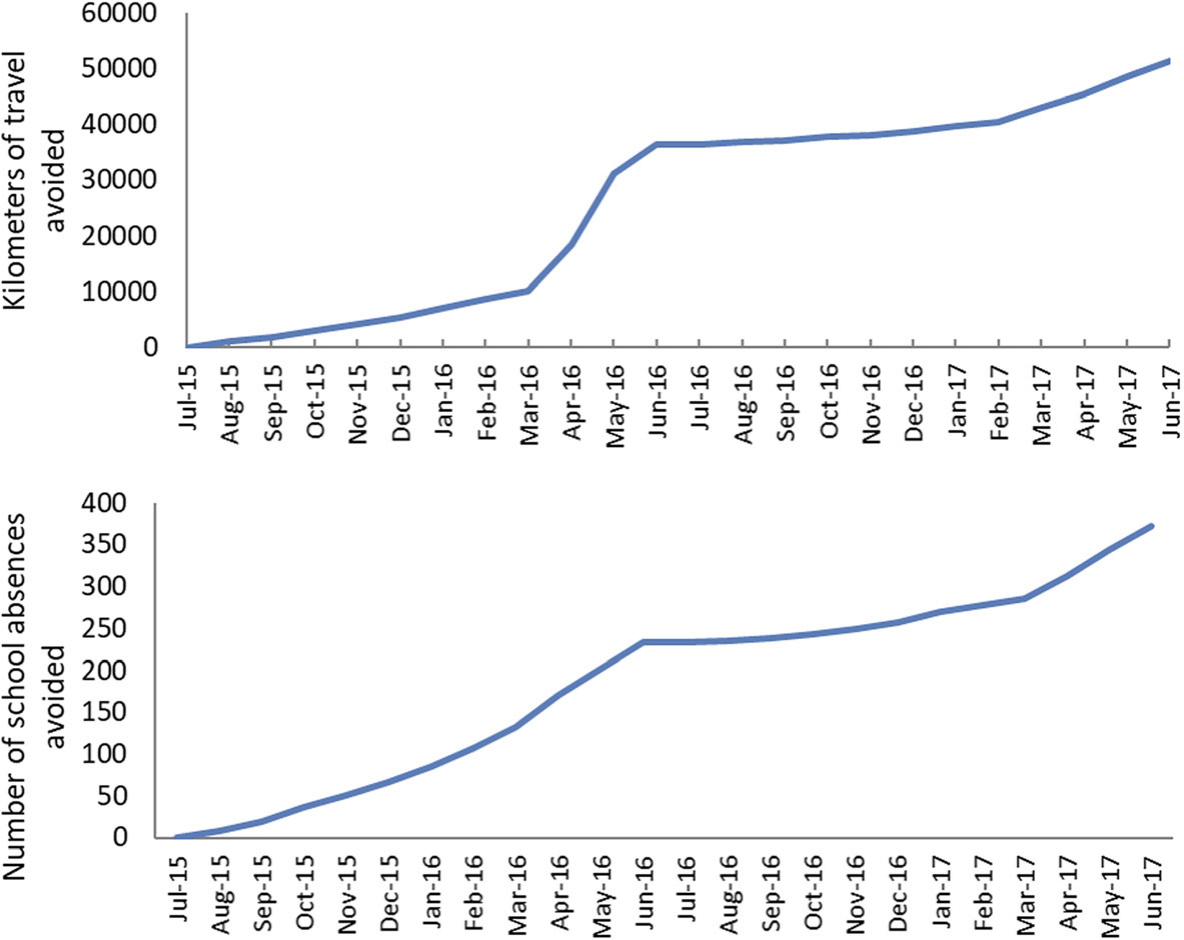
Estimated cumulative travel distances avoided by families whose children were enrolled Care Coordination

Based on data collected by Care Coordinators on prevented hospital encounters over 2 years, an estimated 51,416 km of travel was saved for families, and 370 school absences were avoided among children aged over 5 years (Fig. 3). The savings for families, on travel costs alone, are estimated at a total of $98,317 based on the whole of vehicle life per kilometre cost estimates and the average cost of petrol per kilometre for a common vehicle.

## Discussion

The implementation of the Kids GPS Care Coordination service at the SCHN has resulted in significant reductions in the number of hospital encounters for enrolled children and associated costs for these hospital encounters. Estimating the number of hospital encounters prevented was a significant challenge. The data collected by the care coordinators on encounters saved for all enrolled children estimated a greater number of prevented hospitalizations than the routinely collected hospital administrative data. This may be because care coordinators were able to count hospitalisations saved for infants aged under 6 months for whom there was no pre- comparison, and they also counted encounters that were imminent but were averted because of Care Coordination, and such encounters could never be collected through the hospital administrative data system. Nevertheless, both estimation methods suggested savings of over Au$4million over 2 years, and our results concur with those of others who also found significant reductions in hospitalisations and ED presentations and associated costs. [3, 5, 22]

The Kids GPS Care Coordination service had a wide reach across SCHN with many different medical teams and care providers involved in the care of enrolled children. General medicine teams were involved most often in the care of children who were enrolled in the service. This is not surprising as general paediatricians often take on the role of providing ongoing care in between appointments with specialist teams. The Care Coordinators have nurtured the development of clinical partnerships among multiple clinical teams involved in the care of children with complex medical needs by attending routine case review meetings and making connection with clinical nurse specialists and other key personnel working with medical teams. These clinical partnerships have built the capacity of specialist medical teams such as gastroenterology and rheumatology to embed the practice of integrating patients locally where possible. Care Coordinators have been proactive in linking families with paediatricians working in local hospitals or in the community to meet the child’s needs closer to home and away from tertiary/quaternary paediatric hospitals. In alignment with the WHO frameworks for patient centered integrated care, to strengthen and extend the role of primary care, [17] the ongoing engagement with GPs who are appropriately skilled, supported and integrated into the circle of coordinated care is a priority to improve delivery of needed services closer to home and for the maintenance of optimum health for children in between regular visits to specialists and paediatric hospitals. The OECD and the WHO have also called for supports that facilitate self-care [7, 17], and linking families in our study with local GPs and the Kids GPS 24-h Hotline aligns with these recommendations. Enabling parents to make decisions while being supported close to home, has been important for ensuring that the paediatric hospital ED is not always the first point of access when care is needed. This also has benefits for families who saved time and money on travel because of the reduced number of visits to the paediatric hospitals. However, the cost savings for families go well beyond the calculated cost of kilometres travelled, and include costs of accommodation and food, cost of care for siblings while the parents or carers are away at the hospital with the sick child, and days away from employment which may result in loss of income. These additional costs borne by families have not been estimated in this evaluation project, and we would recommend that data on these aspects be collected during the next evaluation phase.

Sharing health information and care plans across all health care providers and with the patient, parent and/or caregiver was an important component of the Care Coordination service. Shared IT infrastructure, e-health records and shared care plans are identified in the literature as priorities for improving quality and safety of health care and patient and care giver experiences [7]. We plan to further improve information sharing and appointment coordination by using a patient-held smart phone app that enables the parent to share the care plan with any provider in any setting if they choose.

The number of new referrals increased considerably in the second year and a large gap between referrals and enrolments emerged, mostly because children that did not meet eligibility criteria were referred. During implementation of the Kids GPS Care Coordination service strategies were put in place to prevent duplication of service so that children who already had a care coordinator in their medical team, or their needs could be better served by another more appropriate service were diverted to other service options. As part of continuous improvement, a new communication strategy is currently being developed to provide medical teams and parents with clear information about the scope of the Kids GPS Care Coordination Service and eligibility criteria for enrolment. This is likely to decrease the number of inappropriate referrals, thereby reducing the Care Coordinators’ workload associated with assessing referred cases for eligibility.

Integration of the health sector with social services and community support services has been shown to reduce health service burden [7]. Addressing the psycho-social needs of CMC is an important and integral part of providing health care and this was a strong priority highlighted during consultations undertaken with health care providers as part of the formative evaluation [25]. The effectiveness of health care provision is inextricably linked with the families’ socio-economic status and capacity. This must be considered when developing the circle of coordination around the individual child and their family [5]. The relatively high proportion of children enrolled in the care coordination service who lived in areas classified as socio-economically disadvantaged [24] was not surprising. The demographic diversity of the population served by the SCHN includes areas with an over-representation of immigrant, refugee, Aboriginal families and other families living with social disadvantage [26]. The coordinators worked with some families to link them with social care organisations. The psycho-social support needs of CMC and their families have been considered and should continue to be considered when planning for future service capacity. As the service evolves and matures, so too the roles of the Care Coordinators are likely to evolve and the number of coordinators needed and their skill-sets may need adjustment.

### Strengths and limitations

Estimating encounters and cost savings using routinely collected administrative data has several limitations. It is impossible to compare pre and post enrolment encounters for newborn infants because there is no pre-enrolment data. However, the Care Coordinators were in a unique position from the outset to prospectively collect data on outcome indicators, particularly encounters prevented or streamlined. The data collected by care coordinators underestimated the number of presentations to ED and the number of day-only admissions saved, however it was a very useful additional data source.

The cost savings are likely to be underestimated because calculations are based on average costs per encounter for all patients across the network and do not take into account actual costs for each individual patient, and the average costs may not reflect the complexity of service required by enrolled children. There are also likely to be ongoing savings for these children until they transition to adult health care at 18 years of age. We have not analysed the relative costs associated with shifting health care from the tertiary center to care delivered closer to home at local hospitals in LHDs, by GPs and community health services. The next phase of evaluation should include an analysis of any increases in demand for services outside of the SCHN hospitals, including LHDs, community health services and primary care. Ongoing longitudinal analysis of administrative data from the MSAU is likely to provide more accurate estimates of the numbers of encounters saved at the SCHN in the future.

## Conclusion

Clear benefits of the Care Coordination service for the tertiary paediatric network and for families have been demonstrated with significant reductions in ED presentations, day only admissions and hospitalisations. Demonstrating outcomes due to health system change is extremely challenging, however we in-built a systematic data collection while developing and implementing the model of care to enable ongoing evaluation and model adjustment. Furthermore, the co-design approach was used when developing the model with engagement of all key partners which supported the implementation and evaluation processes and enabled management of expectations from stakeholders. Plans to develop and implement a wide-ranging and multi-faceted communication strategy will further support engagement with the SCHN, LHDs, community and primary health providers, to build even better relationships that support the ongoing work of the Care Coordination service. Future evaluation waves are planned and are essential for continuous optimisation of the model of care.

## Abbreviations

CHW: Children’s Hospital at Westmead
CMC: Children with Medical Complexity
ED: Emergency Department
FTE: Full Time Equivalent
GP: General Practitioner
HREC: Human Research Ethics Committee
IRSAD: Index of Relative Socio-Economic Advantage and Disadvantage
Kids GPS: Kids Guided Personalised Service
LHD: Local Health District
LNR: Low and Negligible Risk
MSAU: Management Analysis and Support Unit
NRMA: National Roads and Motorists Association
NSW: New South Wales
OECD: Organisation for Economic Cooperation and Development
PHN: Primary Healthcare Network
SCH: Sydney Children’s Hospital
SCHN: Sydney Children’s Hospitals Network
UK: United Kingdom
US: United States of America
WHO: World Health Organisation

## Glossary

Children with Medical Complexity (CMC): Children with family-identified service needs, characteristic chronic and severe conditions, functional limitations, and high health care use of a type or amount beyond that required by children generally [5].
Care coordinator: A specialist nurse who facilitates access to health services for the child and family, links families with services closer to home where possible, facilitates care plans that are shared across health teams, health care settings and with families, facilitates access to eHealth supports that enables self-care in the community, and facilitates access to social care and peer support organisations.
General Practitioner: A doctor working in the primary care setting, either in a privately owned solo or group practice facility or in a community health centre run by a Local Health District.
Local Health District (LHD): A local hospital network in New South Wales, Australia, which forms an organisation that provides public hospital services in accordance with the Australian National Health Reform Agreement. A local hospital network can contain one or more hospitals, and is usually defined as a business group, geographical area or community. Every Australian public hospital is part of a local hospital network [6].
